# The *Candida albicans*-Specific Gene *EED1* Encodes a Key Regulator of Hyphal Extension

**DOI:** 10.1371/journal.pone.0018394

**Published:** 2011-04-07

**Authors:** Ronny Martin, Gary P. Moran, Ilse D. Jacobsen, Antje Heyken, Jenny Domey, Derek J. Sullivan, Oliver Kurzai, Bernhard Hube

**Affiliations:** 1 Department of Microbial Pathogenicity Mechanisms, Leibniz Institute for Natural Product Research and Infection Biology – Hans Knoell Institute (HKI), Jena, Germany; 2 Group Fungal Septomics, Center for Innovation Competence Septomics, Research at the Leibniz Institute for Natural Product Research and Infection Biology – Hans Knoell Institute (HKI), Jena, Germany; 3 Friedrich Schiller University, Jena, Germany; 4 Microbiology Research Unit, Division of Oral Biosciences, School of Dental Science and Dublin Dental University Hospital, University of Dublin, Trinity College Dublin, Dublin, Ireland; University of Aberdeen, United Kingdom

## Abstract

The extension of germ tubes into elongated hyphae by *Candida albicans* is essential for damage of host cells. The *C. albicans*-specific gene *EED1* plays a crucial role in this extension and maintenance of filamentous growth. *eed1*Δ cells failed to extend germ tubes into long filaments and switched back to yeast growth after 3 h of incubation during growth on plastic surfaces. Expression of *EED1* is regulated by the transcription factor Efg1 and ectopic overexpression of *EED1* restored filamentation in *efg1*Δ. Transcriptional profiling of *eed1*Δ during infection of oral tissue revealed down-regulation of hyphal associated genes including *UME6,* encoding another key transcriptional factor. Ectopic overexpression of *EED1* or *UME6* rescued filamentation and damage potential in *eed1*Δ. Transcriptional profiling during overexpression of *UME6* identified subsets of genes regulated by Eed1 or Ume6. These data suggest that Eed1 and Ume6 act in a pathway regulating maintenance of hyphal growth thereby repressing hyphal-to-yeast transition and permitting dissemination of *C. albicans* within epithelial tissues.

## Introduction

*Candida albicans* is normally a harmless commensal and part of the microflora on mucosal surfaces, but frequently causes superficial infections such as oral or vaginal thrush. Infection of epithelial surfaces is associated with extensive growth, invasion into and dissemination within epithelial tissues and inflammation. In some circumstances, for example after organ transplantation, the fungus may cause invasive and life threatening systemic infections. In these patients, the fungus can disseminate, usually from the gastrointestinal tract or from biofilms on medical devices, via the bloodstream leading to invasion of organs such as the liver or kidney [1].

Several virulence attributes of *C. albicans* are considered to play roles during invasion of human cells. One of the most important invasive properties of *C. albicans* is the ability to change growth morphology from spherical yeast cells to elongated hyphae (dimorphism). Hyphae are not only essential for invasion [2], but are also more adhesive to host cells as compared to the yeast growth form [3]. Attachment of *C. albicans* hyphae to epithelial surfaces is mediated by hyphal-associated ÿnvasionÿ, namely the glycosylphosphatidylinositol (GPI)-anchored protein Hwp1 [4] and members of the GPI-anchored agglutinin-like Als protein family of ÿnvasionÿ [5]. To invade non-phagocytic cells, such as epithelial cells, fungal hyphae either actively penetrate host cells or trigger up-take via induced endocytosis by host cells [6]–[8]. The route of invasion depends on the host cell type. For example, invasion of oral epithelial cells occurs via induced endocytosis and active penetration, while enterocytes are only invaded by active penetration [8]. The Als family member Als3 acts as a *C. albicans* _nvasion triggering fungal up-take via interactions with receptors on epithelial or endothelial cells [9]. Several other hyphal- associated factors may have specific functions during attachment and invasion of host cells [10], [11]. After initial invasion into superficial epithelial cells, fungal hyphae can penetrate into deeper cell layers and disseminate, as shown previously for oral epithelial tissue [12].

Many studies have investigated the environmental conditions, which induce the yeast-to-hyphal transition of *C. albicans in vitro*. Known inducers of hyphal formation are a temperature shift to 37°C, addition of serum, the increase of environmental pH to physiological values between pH 6 and 7, hypoxic conditions, physiological CO_2_ concentrations or contact with surfaces ranging from plastic (e.g. catheters) to human host cells [13]–[15]. Furthermore, ingestion by macrophages can induce hyphal growth of *C. albicans* as part of an escape mechanism: phagocytosed yeast cells can produce hyphae, which pierce the host membrane and kill the macrophage [16], [17]. In addition, the transition between yeast and hyphal growth forms has a significant effect on the host immune response and possibly on the outcome of an infection [18], [19]. Therefore, hyphae seem to be not only essential for adhesion and invasion, but also for immune evasion. Consequently, nonfilamentous mutants are strongly reduced in virulence [16]. However, hyperfilamentous mutants which lack the hyphal repressors Nrg1 or Tup1 and which cannot grow in the yeast form are also reduced in virulence [20], [21]. This suggests that morphological plasticity and the ability to grow either in the yeast or the hyphal phase are essential for virulence of *C. albicans*. Due to the importance of the yeast-to-hyphal transition for virulence of *C. albicans*, multiple studies have investigated the molecular and cellular events associated with this morphological transition. Signal transduction pathways controlling hyphal formation such as the mitogen- activated protein (MAP-) kinase cascade and the cAMP pathway converge at the transcription factors Cph1 and Efg1 which together are crucial for hyphal formation and the activation of hyphal-associated genes [14], [15],[22]. Other cellular factors such as the Rho-GTPase Cdc42 and the cyclin Hgc1 play further important roles in the regulation of filamentous growth of *C. albicans*. Hgc1 has been shown to phosphorylate Efg1 and mutants lacking Hgc1 fail to form true hyphae, but still express certain hyphal-associated genes [23]–[25]. Although much is known about hyphal induction of *C. albicans*, regulation of hyphal extension is less well studied and despite intensive research on dimorphism, it is still poorly understood how the switch from hyphal-to-yeast growth is regulated. Only a few genes, such as *PES1*, which encodes a *C. albicans* pescadillo homolog, were shown to be involved in the switch from hyphae to yeast cells [26]. Furthermore, the consequences of a dimorphic switch on pathogenesis remain unclear. More recent studies have identified another key transcription factor, Ume6, which is necessary for the extension of germ tubes into hyphae [27]. Overexpression of *UME6* can restore filamentation in several mutants which are unable to form hyphae [28].

We recently identified a *C. albicans* gene of previous unknown function, which was expressed during oral tissue infections and in patients suffering from oral infections. Mutants lacking this gene were able to invade superficial oral epithelial cells, but once inside a host cell, the mutants grew as yeast cells, remained trapped intracellularly and did not disseminate within epithelial tissue. Therefore, the gene was named *EED1* (Epithelial Escape and Dissemination 1) [12].

Here, we show that Eed1 is a unique protein of *C. albicans* and essential for hyphal extension on solid surfaces and during interaction with host cells. Expression of *UME6* depends on Eed1, which itself is a target of the transcription factor Efg1, and ectopic overexpression of *UME6* restored hyphal elongation in *eed1*Δ. We suggest that Eed1 and Ume6 act in a pathway which controls the extension of germ tubes into hyphae, the hyphal-to-yeast transition and escape from non-phagocytic host cells.

## Results

### *EED1* is unique to *C. albicans*

Using Blast searches within the available genomic sequences we aimed to identify homologues of *EED1*. The closest hit was *DEF1*, coding for a regulator of RNA polymerase II (RNAPII) with multiple functions in *S. cerevisiae* [12], [29], [30]. In fact, Eed1 has structural similarities with Def1: both proteins are of comparable length and unusually rich in glutamine residues over a 200–300 amino acid central region [12]. However, we found the overall identity (13%) and similarity (18.2%) were low and no homology was found flanking the glutamine rich region, suggesting that both genes have evolved independently and are likely to have different functions. No homolog of *EED1* was detected in the genomes of species within the CUG clade, including very close relatives of *C. albicans* ([31]). This was despite the fact that the gene locus of *EED1* in *C. albicans* (containing genes *YTA6*, *BET2*, *DPB2*, and *GPN3*) is conserved within the CUG species, although the gene order differs (not shown). The closest relative of *C. albicans*, *C. dubliniensis*, contains a syntenic gene between *YTA6* and *BET2* named *MDP1* (Moran et al., unpublished data). However, the overall identity between Eed1 and Mdp1 is low (13.4%), with a slightly higher similarity (26.6%) than between Eed1 and Def1. These data suggest that Eed1 is unique to *C. albicans*.

### Dynamics of transient filamentous growth of *eed1*Δ on plastic surfaces

Mutants lacking *EED1* were unable to produce true hyphae in liquid media and only transiently produced filaments during contact with oral epithelial cells, but switched to yeast cell growth during the infection process [12]. Induction of hyphal formation of *eed1*Δ during co-cultivation with epithelial cells was dependent on contact with epithelial cells, but also occurred after contact with other surfaces (e.g. plastic). To study the dynamics of this contact-dependent, transient filamentation of *eed1*Δ cells in more detail, we analyzed growth of wild type and mutant cells on plastic surfaces in RPMI medium via time lapse microscopy.

Since the triple auxotrophic wild type strain BWP17 was used to produce the *eed1*Δ mutant [12] and since differences between the parental *C. albicans* wild type SC5314 and the derivative BWP17 are sometimes observed, we compared these two strains in all assays used in this study. For comparison, we used an autotrophic version of BWP17 carrying the plasmid pCIP30 (see Material and Methods). Both strains behaved similarly in all assays and no differences were observed. Therefore, we used strain SC5314 in all further experiments as a wild type control.

In addition to the *eed1*Δ mutant described in Zakikhany et al. [12] we also have produced an *eed1*Δ mutant with SC5314 as a parental strain. Regardless of the strain background, either BWP17 or SC5314, *eed1*Δ mutants showed similar phenotypes throughout this study (data not shown). The growth rate of *eed1*Δ mutant and wild type yeast cells was similar (not shown).

**Figure 1:**
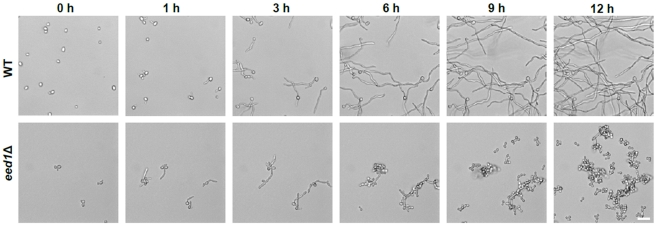
Dynamics of *C. albicans* wild type and *eed1*Δ during growth on plastic surfaces. *C. albicans* wild type (WT) or *eed1*Δ mutant cells were grown in RPMI1640 medium on Petri dishes at 37°C and 5% CO_2_. Growth was monitored by timelapse microscopy with a ZEISS AxioObserver. Z1 microscope and analyzed by Axiovision software. Selected pictures from movies S1 and S2 are shown here. Scale bar: 20 µm.

Supplemental movies S1 and S2 and Fig. 1 clearly show that both wild type and mutant cells respond to contact to plastic surfaces in RPMI medium by forming germ tubes. However, after 3 h, wild type germ tubes continued to extend and occasionally showed branching filaments, while *eed1*Δ germ tubes did not form such structures. Instead we observed budding of yeast cells from the initial filaments (“budding filaments”) (Supplemental movies S1 and S2̧ Fig. 1). Over time, the wild type strain maintained hyphal growth and produced a dense mycelium at time point 12 h. In sharp contrast, the entire population of *eed1*Δ cells grew as yeast cells after 12 h (Fig. 1, 12 h). These morphological differences were accompanied by dramatic differential adhesion properties. While germ tubes of wild type and *eed1*Δ cells adhered to the plastic surface, budding filaments almost completely lost their adherence properties and yeast cells were released from the surfaces (Supplemental movies S1 and S2). These altered phenotypes of *eed1*Δ cells indicate not only differences in the morphology, but also in the expression of hyphal associated adhesins.

### Analysis of septation and budding events in primary *eed1*Δ filaments

**Figure 2:**
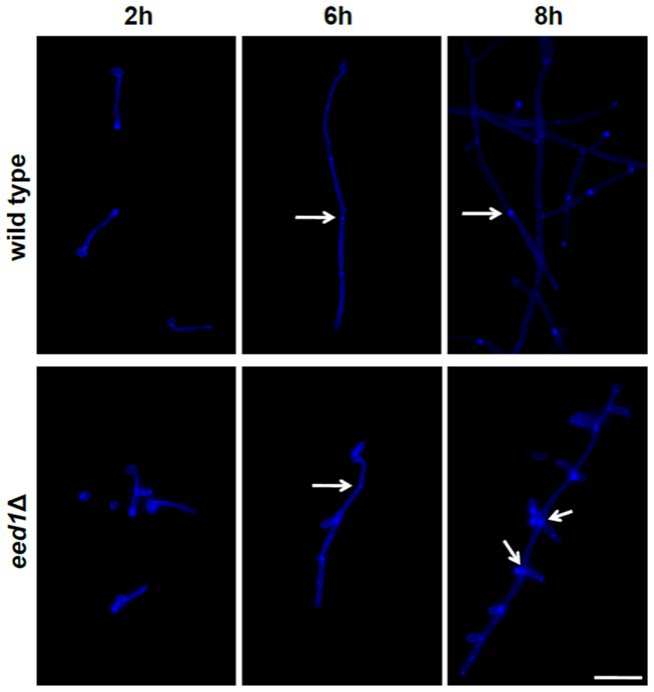
Analysis of septation in *C. albicans* during growth on glass surfaces. *C. albicans* wild type and *eed1*Δ cells were grown on glass slides (37°C, RPMI1640 medium) for the indicated incubation times. After incubation, cells were fixed with 4% Histofix, washed with 1x PBS and stained with Calcofluor White prior to microscopy. Septae are indicated by arrows. Scale bar: 20 µm.

To further analyze the cellular morphology and dynamics of “budding filaments” of *eed1*Δ as compared to wild type cells, we questioned whether primary filaments were able to produce septae. In order to stain septae with Calcofluor White, wild type and mutant cells were grown on glass surfaces with RMPI1640 medium at 37°C. As shown in Figure 2, both wild type and *eed1*Δ cells formed filaments with septae (Fig. 2, 6 h, septae marked by arrows). Budding of *eed1*Δ yeast cells from primary filaments occurred predominantly from sites of septation (Fig. 2, 6 h, 8 h, marked by arrows). These results suggest, that septum formation is not affected by the deletion of *EED1*, that true hyphae are formed after contact to surfaces and that budding of yeast cells from filaments occurs after septation.

### Identification of genes regulated by *EED1*

**Figure 3:**
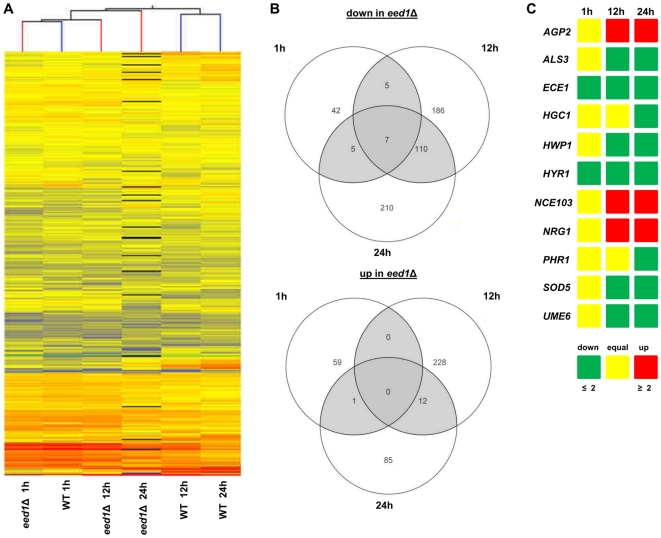
Transcriptome analysis during experimental oral epithelial tissue infections. (A) Global clustering of transcriptional profiles of *C. albicans* wild type (WT) and *eed1*Δ during RHE infections over a time period of 24 h. (B) The numbers of genes differentially expressed at least with a 2-fold change are shown in Venn diagrams for all three points (1 h, 12 h, 24 h). (C) Dynamics of the expression of selected morphology-associated genes in WT and *eed1*Δ during the time course, shown by the ratio between expression in *eed1*Δ and expression in wild type.

To identify genes whose expression is influenced by the activity of *EED1* during interaction with host epithelial cells, we analyzed the genome wide gene expression profiles of both wild type and *eed1*Δ mutant cells during an experimental oral tissue infection using reconstituted human oral epithelium (RHE). Total fungal RNA was isolated 1 h, 12 h and 24 h after infection, labeled and hybridized to *C. albicans* DNA microarrays. Clustering of all transcriptomes during RHE infections revealed that wild type and *eed1*Δ transcript profiles clustered closely together at the earliest time point (1 h), reflecting the similar morphologies and pathogenic interactions with host cells (Fig. 3 A). However, at time points 12 and 24 h, profiles for the *eed1*Δ strain form a subcluster, which is more related to the 1 h time point of *eed1*Δ than to the transcriptional profiles of wild type cells at 12 and 24 h (Fig. 3 A). One hour after infection, only 59 genes were at least 2-fold up-regulated in wild type compared to *eed1*Δ, while 60 other genes were up-regulated in the mutant compared to the wild type (Fig. 3 B). Multiple genes associated with filamentous growth were similarly expressed in both wild type and *eed1*Δ, for example *HWP1*, *CHT2* and *ALS3* or the transcription factors *CPH2*, *UME6* and *TEC1* (Fig. 3 B, C). However, the global differences in expression between wild type and *eed1*Δ increased over time. Twelve h after infection, 548 genes were at least 2-fold differentially regulated in both strains (308 genes up-regulated in wild type, 240 up-regulated in *eed1*Δ, Fig. 3 B). This trend continued up to the 24 h time point. Out of 430 differentially regulated genes, 332 were up-regulated in wild type and only 98 were up-regulated in mutant cells (Fig. 3 B). No gene was up-regulated in the mutant at all time points, however, seven genes were always up-regulated in the wild type. Among these were the hyphae-associated genes *ECE1* and *HYR1* (Fig. 3 A, B). Other hyphal-associated genes, which were similarly expressed in wild type and mutant cells 1 h after infection, were down-regulated in the mutant at later time points, for example *ALS3*, *HWP1* and *SOD5* (Fig. 3 C). The pH response gene *PHR1* was down-regulated in the mutant after 24 h (Fig. 3 C). Furthermore, the transcription factor genes *CPH2*, *TEC1* (not shown) and *UME6* or *RDI1*, involved in polarized growth, were down-regulated in *eed1*Δ after 12 and 24 h (Fig. 3 C, Table S1), with *UME6* being down-regulated 4-fold in the mutant after 24 h. Other genes, such as *WOR2*, involved in the regulatory circuit that controls white-opaque switching, were up-regulated in *eed1*Δ after 12 and 24 h (Table S2). Some genes associated with yeast-like growth were up-regulated in *eed1*Δ at later time points. Among these are the hyphal growth repressor gene *NRG1*, which was 2.4-fold up-regulated in *eed1*Δ 24 h after infection (Fig. 3 C) and the amino acid permease gene *AGP2* which is up-regulated in *eed1*Δ 12 and 24 h after infection (Fig. 3 C, Table S2).

### Expression of the regulatory gene *UME6* depends on Eed1

**Figure 4:**
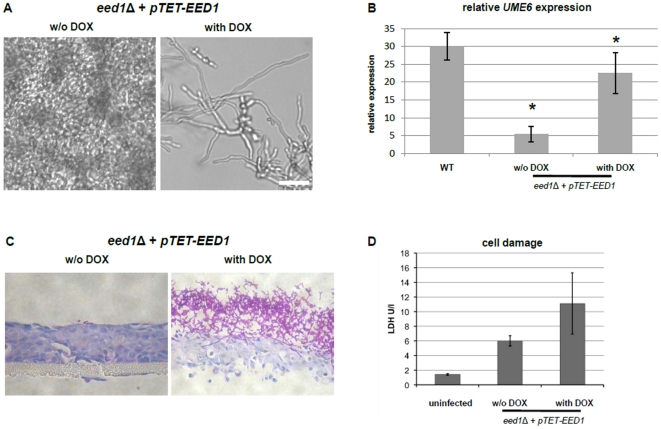
Expression of the *UME6* gene depends on *EED1* expression levels. (A) *C. albicans eed1*Δ mutant cells harboring the ectopic *pTET-EED1* were grown on plastic (RPMI1640 medium, 37°C, 5% CO_2_) for 12 h either in the absence or presence of 50 µg/ml doxycycline (with or without DOX) in the medium. Scale bar: 20 µm. (B) Expression of the *UME6* gene was down- regulated in *eed1*Δ compared to wild type. Expression was restored after overexpression of *pTET-EED1*. Expression was detected by qRT PCR using SYBR Green. Gene expression was normalised against *EFB1* and *ACT1*. Total RNA was isolated after 12 h growth on plastic surfaces. Asterisk means p≤0.05. (C) Infection of reconstituted human oral epithelium (RHE, Skinethic) was carried out using the *eed1*Δ mutant harboring the ectopic *pTET-EED1* cassette. Induction of *EED1* expression was achieved by the addition of 50 µg/ml doxycycline to the infection. Pictures show PAS stained sections of the RHE tissues. (D) Average LDH release caused by *eed1*Δ + *pTET-EED1* exhibited in the presence and absence of doxycycline in three independent infections compared to uninfected RHE tissue.

Since *eed1*Δ cells showed a strong and significant down-regulation of *UME6*, recently shown to be necessary for the extension of germ tubes into hyphae [27], we focused further experiments on the genetic interactions between *UME6* and *EED1*. In order to analyze the influence of *EED1* on the expression level of *UME6* in more detail, we quantified the expression of *UME6* and monitored morphology during growth on plastic surfaces in a *C. albicans* wild type strain and an *eed1*Δ mutant strain carrying a *pTET-EED1* construct at the *ADH1* locus. After addition of 50 µg/ml doxycycline, which activated the tetracycline promoter driven *EED1* allele, the mutant strain grew as filaments and expressed *UME6* at significantly higher levels as compared to control cells in the absence of doxycycline (Fig. 4 A, B). *pTET*-driven expression of *EED1* during infection of RHE restored filamentation and the ability to cause cell damage of the *eed1*Δ mutant (Fig. 4 C, D). These results indicate that expression of *UME6* depends on the expression of *EED1* and that the morphological defects of *eed1*Δ cells may be caused by a down-regulation of *UME6* expression.

### Ectopic overexpression of *UME6* rescues filamentation in *eed1*Δ

Since *UME6* was found to be down-regulated in *eed1*Δ, we hypothesized that *UME6* may be a downstream target of Eed1 and that overexpression of *UME6* in *eed1*Δ may rescue the lack of hyphal extension in *eed1*Δ. Therefore, we integrated a *pTET-UME6* construct into the *ADH1* locus of the *eed1*Δ mutant and analyzed the phenotype of *eed1*Δ after forced expression of *UME6*.

**Figure 5:**
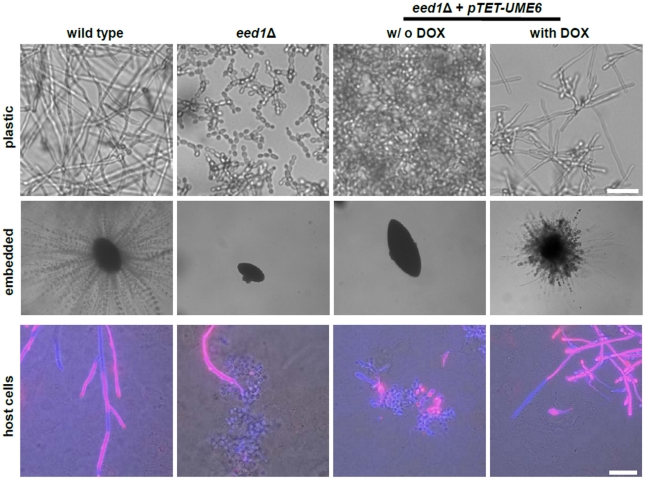
Ectopic overexpression of *UME6* restores filamentous growth in *eed1*Δ. *C. albicans* wild type or *eed1*Δ mutant cells were incubated for 12 h on plastic surfaces (37°C, RPMI1640 medium, 5% CO_2_) or embedded in YPS agar and agar plates were incubated for 3 days at 25°C prior to microscopy. Additionally, wild type and *eed1*Δ were co- incubated with human oral epithelial cells (TR 146 cell line, DMEM medium, 37°C, 5% CO_2_) for 24 h. Those parts of fungal cells, which were outside of host cells, were stained with Anti-Ca 560 (red) and Calcofluor White (blue) resulting in a purple overlay. Those parts, which were inside the host cells, were stained with Calcofluor White only and appeared blue. To induce expression of the tetracycline promoter 50 µg/ml doxycycline were added (indicated as with DOX). Scale bar: 20 µm.

Growth of wild type, *eed1*Δ and *eed1*Δ + *pTET-UME6* cells was tested under three different conditions: (a) growth on plastic, (b) growth under embedded conditions and (c) during infection of oral epithelial cells (TR- 146 cell line). During growth on plastic and during co-incubation with host cells, wild type cells formed hyphae while the *eed1*Δ mutant grew as yeast cells after initial filamentation (Fig. 5). In contrast to the wild type, cells lacking *EED1* failed to produce any filaments under embedded conditions, showing that Eed1 is also required for filamentation under these conditions (Fig. 5). However, forced overexpression of *UME6*, within *eed1*Δ cells caused filamentation under all three conditions tested (Fig. 5).

### Identification of Ume6- regulated gene subsets in *eed1*Δ

**Figure 6:**
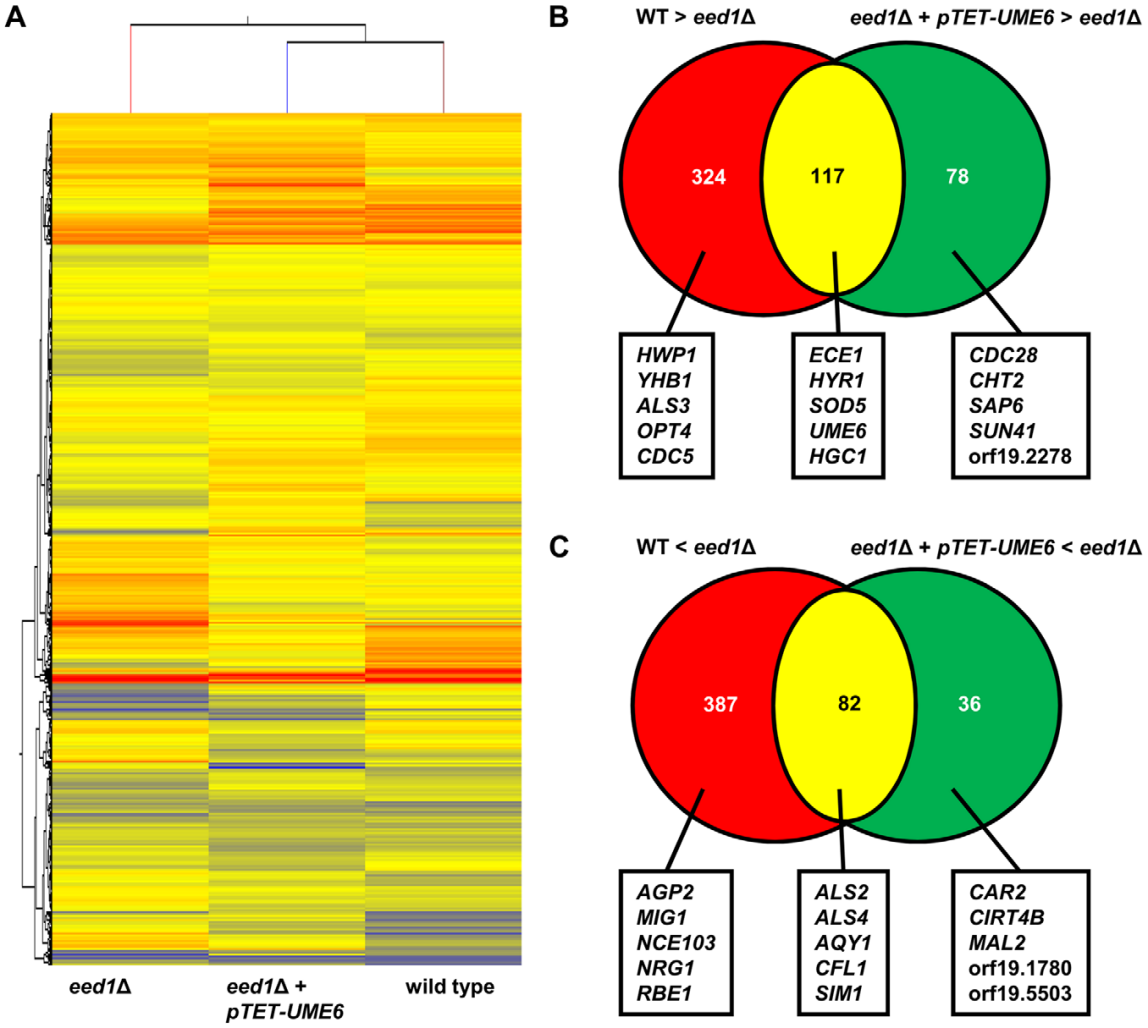
Transcriptome analysis of the effects of ectopic overexpression of *UME6* in *eed1*Δ during growth on plastic. (A) Global clustering of transcriptional profiles of *C. albicans* wild type (WT), *eed1*Δ and *eed1*Δ expressing *pTET-UME6* after 12 h growth on plastic surfaces. (B) Venn diagram showing the numbers of genes at least 2 fold down-regulated in *eed1*Δ compared to WT (red and yellow) and in *eed1*Δ compared to *eed1*Δ + *pTET-UME6* (yellow and green). The intersection (yellow) indicates the number of genes whose expression is restored by ectopic expression of *UME6* in *eed1*Δ. Selected genes referred to in the text are highlighted. (C) The numbers of genes at least 2 fold up-regulated in *eed1*Δ compared to WT (red and yellow) or *eed1*Δ compared to *eed1*Δ + *pTET-UME6* (green and yellow). The intersection (yellow) indicates the number of genes whose expression is repressed by ectopic expression of *UME6* in *eed1*Δ. Selected genes referred to in the text are highlighted.

Since overexpression of *UME6* in *eed1*Δ restored filamentation during growth on plastic surfaces, we analyzed the transcriptome of wild type, *eed1*Δ and *eed1*Δ + *pTET-UME6* cells with or without doxycycline after 12 h growth on plastic (Fig. 6, Table S3 and S4) to identify genes regulated by Eed1 and/or Ume6. When comparing the transcriptome of wild type and *eed1*Δ cells, we identified 910 genes, which were at least 2-fold differentially expressed in the mutant. Of these, 441 were down-regulated and 469 were up-regulated in *eed1*Δ (Fig. 6, Table S3). Compared to *eed1*Δ, 313 genes were at least 2-fold differentially expressed after forced *pTET-UME6* expression (Fig. 6, Table S4). Of these, 195 genes were up-regulated and 118 genes were down-regulated compared to *eed1*Δ (Fig. 6, Table S4). Clustering of gene expression pattern of wild type, *eed1*Δ and *eed1*Δ + *pTET-UME6* cells showed that wild type and *eed1*Δ + *pTET-UME6* gene expression pattern clustered together (Fig. 6A), a result which reflected the observed similar morphologies (Fig. 5). Among the 441 genes, which were down-regulated in the *eed1*Δ mutant on plastic (Table S3), were several hyphae-associated genes such as *ECE1*, *HYR1*, *HWP1* and *SOD5* and regulatory genes such as *UME6* and *HGC1* (Fig. 6 B). All of them were also down-regulated in the *eed1*Δ mutant during RHE infections (Fig. 3). Ectopic overexpression of *UME6* in *eed1*Δ could restore the expression of 117 of these 441 down-regulated genes to wild-type levels, including *ECE1*, *HYR1*, *SOD5*, *HGC1* and *UME6* (Fig. 6 B, Table S4). The remaining 324 genes did not reach wild type levels in the microarray analysis. However, 78 genes were expressed even higher in the *eed1*Δ + *pTET-UME6* mutant as compared to the wild type (Fig. 6 B, Table S4). Among these were filament-associated genes like *SAP6* and *SUN41*.

Among the 469 genes which were up-regulated in *eed1*Δ during growth on plastic as compared to the wild type were yeast-specific genes such as *AGP2*, regulatory genes like *NRG1* and *MIG1* and the adhesion genes *ALS2* and *ALS4* (Fig. 6 C, Table S3). Some of these expression patterns (e.g. *AGP2*, *NRG1*) reflected the yeast cell growth of *eed1*Δ at the 12 h time point and were similar to the expression pattern monitored during the RHE infection (Fig. 3). However, only 82 out of the 469 genes up-regulated in *eed1*Δ cells were significantly down-regulated to wild type levels following ectopic overexpression of *UME6* (Table S4), including *ALS2*, *ALS4* and *AQY1*. The expression levels of 387 genes, including *NRG1* and *AGP2*, remained unaffected by overexpression of *UME6* in *eed1*Δ.(Fig. 6 C). Thirty six genes exhibited greater down-regulation in *eed1*Δ + *pTET-UME6* as compared to wild type levels. Most of these genes were of unknown function including orf19.1780 and orf19.5503 (Fig. 6 C). These results show that a significant portion of *EED1*-regulated genes, including the most prominent hyphae-specific genes *ECE1*, *SOD5* and *HYR1,* the cyclin gene *HGC1* and the hyphal repressor gene *NRG1*, are regulated via *UME6*.

### *EED1* is up-regulated in *C. albicans nrg1*Δ and *tup1*Δ

**Figure 7:**
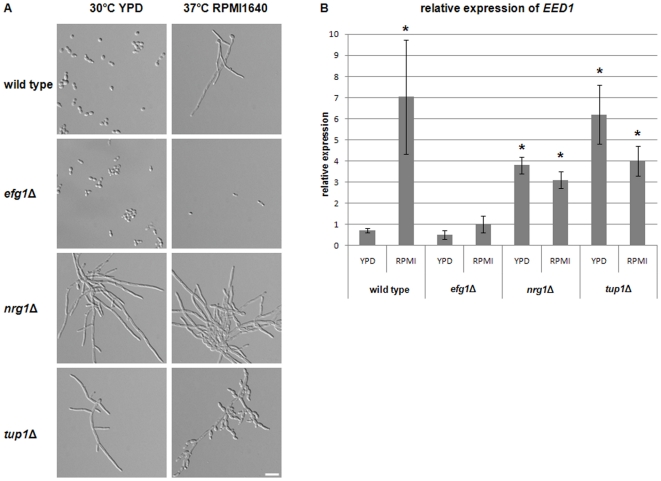
*EED1* expression is regulated by Nrg1, Tup1 and Efg1. (A) Morphology of *C. albicans* wild type, *nrg1*Δ, *tup1*Δ and *efg1*Δ strains at 30°C and 37°C. Strains were incubated in liquid cultures at 30°C for 6 h in YPD or for 6 h in RPMI1640 at 37°C prior to microscopy. Scale Bar: 20 µm. (B) Expression of *EED1* in *C. albicans* wild type and mutant strains under conditions which favor yeast growth (YPD) and hyphal-inducing conditions (RPMI). Expression levels were quantified by qRT PCR using SYBR Green and normalised against housekeeping genes *EFB1* and *ACT1*. Asterisk means p≤0.05.

In order to gain more information about the possible pathways which may be associated with *EED1* regulation, we analyzed the expression of *EED1* in mutants lacking key regulators of hyphal formation. The intergenic region between the open reading frame of *EED1* and the 5′ upstream gene *YTA6* is unusually long consisting of approximately 3 kb. Within this untranslated region we identified three putative Nrg1 response elements (NRE, Table S5,[21]), which suggest a possible regulation of *EED1* expression by this repressor. In fact, under conditions which favor yeast growth of wild type cells (YPD, 30°C, Fig. 7 A), *EED1* was significantly up-regulated in *nrg1*Δ cells as compared to the wild type (Fig. 7 B). Even higher up-regulation of *EED1* was monitored in *tup1*Δ mutant cells at 30°C (Fig. 7 B). Both *nrg1*Δ and *tup1*Δ mutant cells grew as filaments under these conditions (Fig, 6 A). Under conditions which favour hyphal formation of *C. albicans* (RPMI1640 medium, 37°C, Fig. 7 A), *EED1* expression increased 10-fold in wild type hyphae compared to yeast cells (Fig. 7 B). In contrast, expression did not further increase and even slightly decreased in *nrg1*Δ mutant cells under conditions that induce hyphal growth in wild type cells (Fig. 7 B). Similar *EED1* expression patterns were monitored in *tup1*Δ cells which reverted from hyphae to yeast cells and pseudohyphae (Fig. 7 A, B). These data suggest that *EED1* is repressed by Nrg1 and Tup1 in wild type yeast cells.

### *EED1* expression depends on Efg1

In addition to NREs, the promoter of *EED1* contains several E box motifs which resemble binding sites of Efg1, the key transcriptional regulator of the cAMP pathway (Table S5, [32]). A previous study has shown that expression of *EED1*, formerly known as *EDT1* (Efg1- dependent- transcript, [33]) was down-regulated in *efg1*Δ mutant cells. In order to quantify expression of *EED1* in the *efg1*Δ mutant, we incubated the *efg1*Δ mutant and wild type cells under hyphal inducing conditions in liquid medium (RPMI) at 37°C. While the wild type formed hyphae under these conditions, *efg1*Δ cells remained in the yeast-like growth phase. We found that *EED1* was approximately 10-fold down-regulated in *efg1*Δ as compared to wild type hyphae (Fig. 7 B). In contrast, the expression level of *EED1* in *efg1*Δ under conditions which favor yeast growth (YPD, 30°C, Fig. 7 A) was similar to wild type cells. These data suggest that *EED1* is a downstream target of Efg1.

### Ectopic overexpression of *EED1* partially rescues filamentation in *efg1*Δ

**Figure 8:**
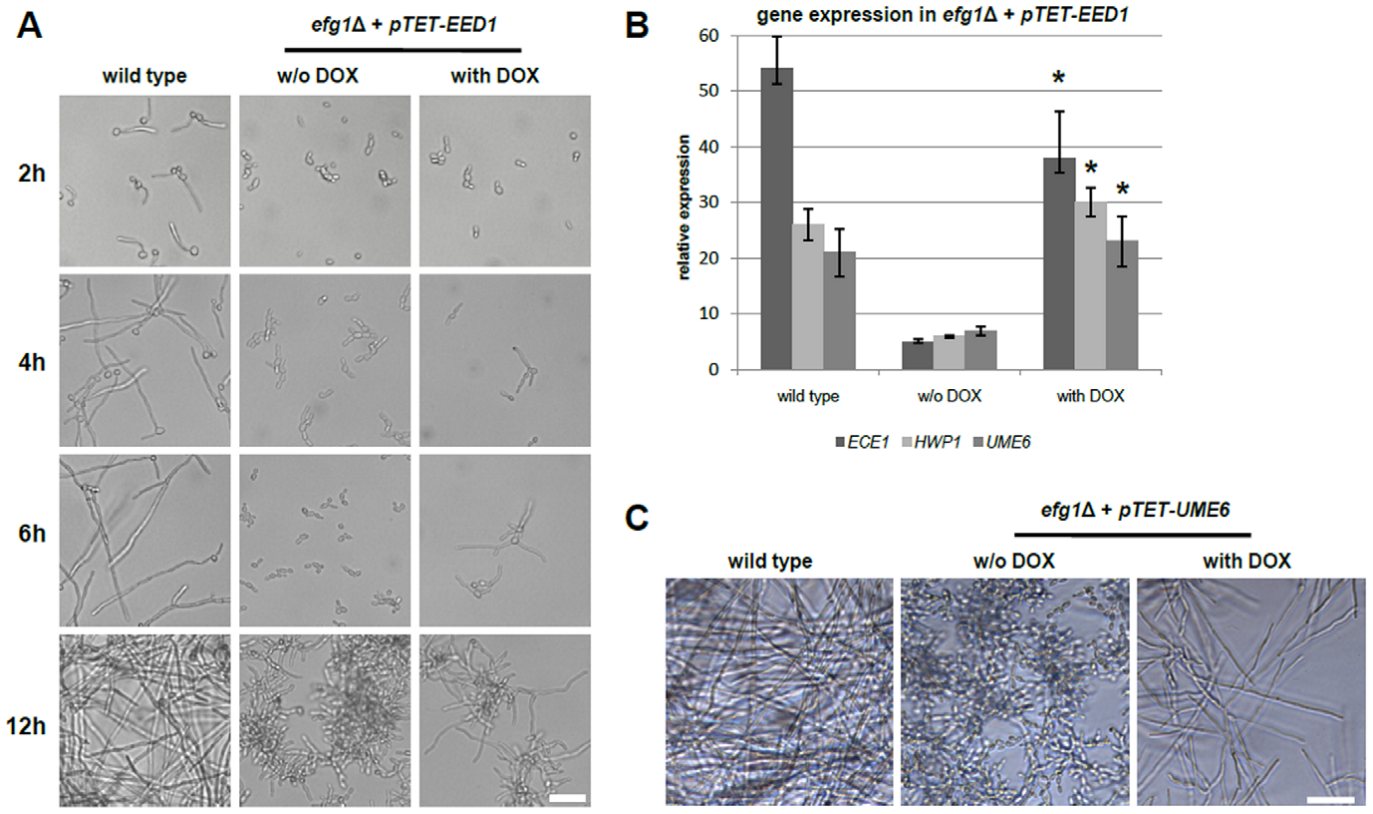
Ectopic overexpression of *EED1* restores filamentation in *efg1*Δ during growth on plastic surfaces. (A) Ectopic overexpression of *pTET-EED1* restored filamentation in *efg1*Δ during 12 h growth on plastic surfaces (37°C, RPMI1640 medium, 5% CO_2_). To induce expression of the tetracycline promoter 50 µg/ml doxycycline were added (indicated as with DOX). (B) Hyphae-associated genes *ECE1*, *HWP1* and *UME6* are upregulated in *efg1*Δ after overexpression of *pTET-EED1*. Expression was detected by qRT PCR using SYBR Green. Gene expression was normalised against *EFB1* and *ACT1*. Total RNA was isolated after 12 h growth on plastic surfaces. Asterisk means p≤0.05. (C) Ectopic overexpression of *pTET-UME6* restored filamentation in *efg1*Δ during 12 h growth on plastic surfaces (37°C, RPMI1640 medium, 5% CO_2_ with or without 50 µg/ml doxycycline). Scale bars: 20 µm.

If *EED1* is a crucial regulator of hyphal extension and a downstream target of Efg1, one would expect that a forced overexpression of *EED1* may cause filamentation in *efg1*Δ. To test this possibility, we ectopically integrated a *pTET-EED1* construct into the *ADH1* locus of the *efg1*Δ mutant and studied morphology of the resulting mutant (*efg1*Δ + *pTET-EED1*) with and without the addition of 50 µg/ml doxycycline during growth on plastic. As expected, wild type strains showed strong hyphal induction on plastic surfaces and *efg1*Δ + *pTET-EED1* failed to form filaments without the addition of doxycycline (Fig. 8 A). Induction of *EED1* by the addition of doxycycline in *efg1*Δ + *pTET-EED1* caused formation of hyphae or pseudohyphae in approximately 80% of all cells after 12 h (Fig. 8 A). Since filamentation of *efg1*Δ mutant cells expressing *EED1* was observed, we analyzed the expression of the hyphal-associated genes *ECE1* and *HWP1* and the hyphal regulatory gene *UME6* in wild type, *efg1*Δ and *efg1*Δ + *pTET-EED1* cells. *ECE1* and *HWP1* were approximately 10-fold down-regulated in *efg1*Δ as compared to the wild type (Fig. 8 B). Forced expression of *EED1* in *efg1*Δ + *pTET-EED1* caused increased expression of *HWP1* similar to wild type levels (Fig. 8 B). Similarly, expression of *ECE1* was increased in *efg1*Δ + *pTET-EED1*, although expression levels were slightly lower as compared to the wild type (Fig. 8 B). For *UME6*, we observed 5-fold reduced expression in *efg1*Δ + *pTET-EED1* cells without addition of doxycycline as compared to the wild type (Fig. 8 B). However, during forced expression of *pTET-EED1* by the addition of doxycycline, the expression of *UME6* increased to the level of wild type hyphae (Fig. 8 B). This supports the hypothesis that *UME6* expression depends on Eed1. Confirming previous observations made by Zeidler et al. [28], we also observed that forced expression of *UME6* restored filamentation in *efg1*Δ cells (Fig. 8 C). It should be noted that hyphal growth in *efg1*Δ + *pTET-UME6* seemed to be stronger than in *efg1*Δ + *pTET- EED1* after addition of doxycycline (Fig. 8 A, C). Taken together, these data suggest that forced expression of *EED1* not only triggered hyphal formation, but also expression of hyphal-associated genes.

## Discussion

### *EED1* is a unique, species-specific gene of *C. albicans*

Although the gene locus of *EED1* is conserved within the ascomycetes CUG clade, we did not find a homologous gene in any genome sequence accessible via NCBI (http://blast.ncbi.nlm.nih.gov/Blast.cgi). Only *C. dubliniensis* contains a sytentic gene at the same locus named *MDP1* (Moran et al., unpublished data). However, the overall homology between Eed1 and Mdp1 is low and functional analysis of Mdp1 in *C. dubliniensis* suggests different roles of both proteins (Moran et al., unpublished data). Therefore, we concluded that *EED1* is a unique gene.

The only putative motif of the deduced protein of *EED1*, which may indicate a cellular function, is a central glutamine-rich region similar to the *S. cerevisiae* protein Def1 [12], [29], [30], which may facilitate interactions with other proteins. In *C. albicans*, many regulatory proteins including Efg1, Cph1, Ume6 and Tec1 possess such multi glutamine stretches (http://www.candidagenome.org, [34], and not shown) suggesting that this glutamine-rich region may have an important regulatory function for Eed1. The intergenic region between *EED1* and the 5′ upstream neighbour gene *YTA6* is unusually long (approximately 3 kb), similar to and typical for other hyphal-associated (e.g. *ALS3*) or hyphal regulator genes (e.g. *EFG1*) [35], [36], which is in agreement with the proposed role of *EED1* in regulation of morphology.

### Eed1 is a key factor within the network regulating dimorphism

**Figure 9:**
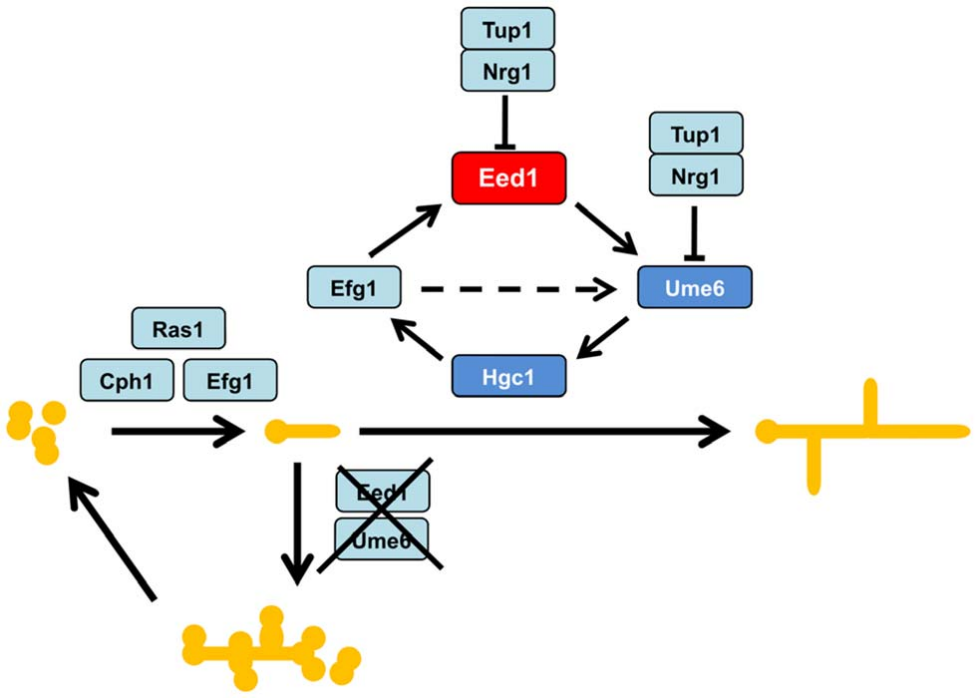
Extension of germ tubes to hyphae depends on Eed1. The proposed model indicates the crucial role of Eed1 during hyphal growth of *C. albicans*. Yeast cells are induced by environmental stimuli to form germ tubes. Regulators like Ras1, Cph1 and Efg1 control this process. Eed1 and Ume6 are essential for extension of initial filaments into long hyphae. The G1 cyclin Hgc1 is regulated by Ume6. Deletion of either *EED1* or *UME6* causes budding of yeast cells from the initial filaments and initiation of hyphal-to-yeast transition.

Due to the importance of *C. albicans* morphology for pathogenicity, multiple studies have investigated the processes involved in hyphal formation and several pathways regulating the morphological transition from yeast-to-hyphal cells have been described [14], [15], [22]. However, the regulation of hyphal extension of primary filaments into long and dividing hyphae is less well studied. Due to the phenotype of cells lacking *EED1*, which were able to form germ tubes but failed to extend these into hyphae with dramatic consequences during infection of epithelial tissues [12], we have focused our study (1) on the regulation of hyphal extension and (2) the gene expression associated with hyphal extension. The results of our work dealing with the regulatory role of Eed1 during dimorphism were combined with recent findings of other studies and summarized in a model shown in Fig. 9. In this model, *C. albicans* cells grow in the yeast form until an external stimulus triggers the formation of germ tubes. This step includes the well- studied MAP kinase cascade and the cAMP pathway as discussed in several reviews [14], [15], [22]. Key regulators of germ tube formation include the GTPase Ras1 and the transcription factors Cph1 and Efg1 (Fig. 9). However, a second regulatory network is required to promote extension of germ tubes into hyphae. When this regulation fails, germ tubes switch back to yeast cells as shown for mutants lacking Eed1 or Ume6 in [12], [27] and in this work (Fig. 9). We propose that Eed1 is the primary element of a regulatory cycle which controls hyphal extension on a transcriptional level. As shown in this work, *EED1* expression depends on Efg1, a member of the APSES gene family [37] (Fig. 9), confirming previous data of the Fink group who originally named *EED1* (orf19.7561) *EDT1* (Efg1-dependent transcript 1) [33]. Expression of *EED1* is also regulated by Nrg1 as shown by transcriptional profiling of the *nrg1*Δ mutant [37] and quantitative RT-PCR (this work) (Fig. 9). The second step of the hyphal extension cycle is an *EED1*- dependent expression of *UME6* (Fig. 9). In this work we provide evidence that expression of *UME6* depends on the expression levels of *EED1*, in particular, mutants lacking either *EED1* or *UME6* share very similar phenotypes [12], [27], supporting our hypothesis that both have important roles in hyphal extension. Similar to *EED1*, *UME6* is repressed by Nrg1 [27], [38] (Fig. 9). It is unclear whether Efg1 directly activates *UME6* expression. We show that *UME6* was down-regulated in *efg1*Δ mutant cells, however, expression of *UME6* was restored by forced overexpression of *EED1*. We suggest that *EED1*-dependent *UME6* expression is an essential step of hyphal extension in *C. albicans*. Overexpression of *UME6* in *eed1*Δ not only restored filamentation, but also expression of prominent hyphal-associated genes like *ECE1*, *HWP1* and *HYR1*. Previously it was shown that *UME6* expression levels have an impact on the growth of *C. albicans* as either pseudohyphae (low *UME6* expression) or true hyphae (high *UME6* expression) [39]. High expression of *UME6* correlated with higher expression of hyphal-associated genes such as *ECE1* and *HWP1* [40], which is supported by our findings. Interestingly, Ume6 seems also be involved in keeping *NRG1* expression levels low in *C. albicans* hyphae [27]. As both, *EED1* and *UME6*, are repressed by Nrg1, this mechanism could contribute to regulating hyphal extension. Our transcriptional data show an up-regulation of *NRG1* in *eed1*Δ during both, infection of RHE and growth on plastic, and this up-regulation was reversed by ectopic overexpression of *UME6* in *eed1*Δ. This may indicate that Eed1-dependent up-regulation of *UME6* is required to keep *NRG1* expression at low levels in order to prevent Nrg1-mediated reversion of hyphae to yeast cells. Another important target of Ume6 seems to be the *HGC1* gene, encoding a hypha-specific G1 cyclin [23], [40] (Fig. 9). *HGC1* was down-regulated in *eed1*Δ at late phases of RHE infections and growth on plastic. This down-regulation was bypassed by ectopic overexpression of *UME6* in *eed1*Δ, indicating that *HGC1* belongs to the set of genes which is regulated by Ume6 as reported previously [40]. Hgc1 itself, together with its interaction partner Cdc28, is involved in the phoshorylation of Efg1 [25], Cdc42 activation [24] and the regulation of polarized secretion [41]. It should be noted, that during growth on plastic, *CDC28* was down-regulated in *eed1*Δ and strongly up-regulated in *eed1*Δ overexpressing *UME6* (Fig. 6). A previous report has shown that mutants lacking another G1 cyclin, Ccn1, were not able to maintain filamentous growth although these cells produced initial germ tubes similar to *eed1*Δ and *ume6*Δ mutant cells [42]. However, in our experiments we did not observe significant changes of *CCN1* expression levels in *eed1*Δ cells suggesting no direct link between Eed1 and Ccn1. Other genes encoding factors involved in polarized growth, including *CDC42*, *RDI1*, *MYO2*, *CDC11*, *CYB2*, *MOB1* and *MLC1* were down-regulated in the *eed1*Δ mutant. Their expression was partially restored to wild type levels after forced overexpression of *UME6* (supplemental tables S3 and S4). These results provide a link between the transcriptional regulation of hyphal extension presented here (Fig. 9) and the known cellular requirements for maintenance of polarized growth and associated structures such as the spitzenkörper. This may explain why mutant cells lacking key elements of the hyphal extension cycle lose their ability to maintain polarized hyphal growth and therefore switch from germ tubes to yeast cell growth. Analysis of the transcriptome data during growth on plastic also showed a slight up-regulation of *PES1* in the *eed1*Δ mutant. This gene was reported to encode a regulator of the hypha-to-yeast switch in *C. albicans* [26] and also plays an important role for dispersion of yeast cells from biofilms [43]. The up-regulation of *PES1* was partially reverted to wild type levels in *eed1*Δ overexpressing *UME6*. This further supports the view that Eed1 and Ume6 are involved in the repression of the reversion of hypha into yeast cells. It was already shown that Ume6 might have a negative influence on dispersion of yeast cells from biofilms [43], [44]. Therefore, it may be postulated that Eed1 also plays a role during biofilm formation.

In summary, our data suggest that Eed1 and Ume6 act in a pathway regulating the maintenance of hyphal growth thereby repressing the hyphal-to-yeast transition.

## Materials and Methods

### *In silico* analysis

The search for homologes of *C. albicans* Eed1 was performed with protein Blast and tBlastn from the NCBI home page (http://blast.ncbi.nlm.nih.gov/Blast.cgi). To compare syntheny for the *EED1* locus within the CUG *Candida* clade we have used the online databases from the Broad Institute (http://www.broadinstitute.org/annotation/genome/candida_group/MultiHome.html) and from the Sanger Institute (http://www.sanger.ac.uk/Projects/Fungi/). Sequences were aligned with DNASTAR Lasergene MegAlign software.

### Strains and media

*C. albicans* strains (listed in Table 1) were routinely grown in YPD medium (1% yeast extract, 2% bacto- peptone, 2% D- glucose) at 30°C or 37°C in a shaking incubator overnight. Prior to use in experiments, fungal cells were semisynchronised by incubating twice in YPD overnight and washed three times with 1x PBS. Cells were counted with a Neubauer chamber and added to experimental assays at the given concentrations. For growth under embedded conditions, cells were grown overnight in YPD medium at 30°C, washed with 1x PBS, diluted to a concentration of 1×10^3^ cells/ml and mixed with YPS agar (1% yeast extract, 2% bacto- peptone, 1% agar, 2% sucrose), plated and incubated for 3 days at 25°C (modified from [45]). Colonies were analyzed with a Leica DM IL inverted microscope (Leica Microsystems, Wetzlar, Germany). Image acquisition and analysis was done with the Leica Application Suite Software.

**Table 1:** *Candida albicans* strains used in this study.

Strain	Genotype	Reference
SC5314	*C. albicans* wild type	[56]
BWP17 + CIp30	*Δura3:: imm434/Δura3:: imm434* *his1::hisG/his1::hisG* *arg4::hisG/arg4::hisG* plus pCIp30	[12]
HLC52(*efg1*Δ)	*efg1::hisG/efg1::hisG-URA3-hisG*in CAI-4	[16]
MMC4 (*nrg1*Δ)	*nrg1::hisG/nrg1::hisG* in CAI-4	[21]
BCA 2-10 (*tup1*Δ)	*tup1::hisG/tup1::hisG* in CAI-4	[20]
UZ149 (*pTET-UME6*)	*ADH1/adh1::SAT1-pTET-UME6*in SC5314	[28]
M1263 (*eed1*Δ)	*eed1::HIS1/eed1::ARG4*, pCIp10in BWP17	[12]
M1273 (*eed1*Δ + *EED1*)	*EED1/EED1* plus pCIp30	[12]
M1315 (*eed1*Δ)	*eed1::FRT/eed1::FRT*in SC5314	this work
M1457 (*tup1*Δ)	BCA 2-10 plus pCIp10	this work
M1458 (*nrg1*Δ)	MMC4 plus pCIp10	this work
M1563 (*eed1*Δ +*pTET-UME6*)	*ADH1/adh1::SAT1-pTET-UME6*in M1263	this work
M1573 (*eed1*Δ +*pTET-EED1*)	*ADH1/adh1::SAT1-pTET-EED1*in M1263	this work
M1574 (*efg1*Δ +*pTET-EED1*)	*ADH1/adh1::SAT1-pTET-EED1*in HLC52	this work
M1764 (*efg1*Δ +*pTET-UME6*)	*ADH1/adh1::SAT1-pTET-UME6*in HLC52	this work
M1773 (*EED1/eed1*)	*EED1/eed1::ARG4, ura3, his1*, pCIp30tin BWP17	his work
M1774 (*eed1/EED1*ΔN)	*eed1::ARG4/EED1ΔN::HIS1, ura3*,pCIp10, in BWP17	this work
M1775 (*eed1/EED1*ΔNQ)	*eed1::ARG4/EED1ΔNQ::HIS1, ura3*,pCIp10, in BWP17	this work
M1776 (*eed1/EED1*ΔQC)	*eed1::ARG4/EED1ΔQC::HIS1, ura3*,pCIp10, in BWP17	this work
M1777 (*eed1/EED1*ΔC)	eed1::ARG4/EED1ΔC::HIS1, ura3,pCIp10, in BWP17	this work

### Cell lines and cell culture

In this study we have used the human oral epithelial cell line TR146 [46]. TR146 cells were grown in DMEM medium with 10% FBS at 37°C and 5% CO_2_ until they have reached confluency. For infection assays, 1×10^5^/ml human cells were plated into 24 well plates and grown in DMEM + 10% FBS at 37°C and 5% CO_2_ until confluency was reached. Prior to invasion, cells were washed and then FBS- free DMEM was added. For infection, *C. albicans* strains were grown in YPD at 30°C overnight and then diluted to OD_600_ = 0.2 in fresh YPD, followed by a new incubation at 30°C for approximately 4 h. Cells were harvested by centrifugation, washed three times with 1x PBS and finally resuspended in 1x PBS. Host cells were infected with fungal cells in different cell numbers ranging from 5×10^2^ (24 h infection) to 1×10^5^ (3 h infection). Infection assays were incubated at 37°C and 5% CO_2_ for a maximum of 24 h.

### Experimental oral epithelial tissue infection

For the analysis of invasion abilities of different *C. albicans* strains we have used the reconstituted human oral epithelium (RHE, SkinEthic, Nice, France) which consists of differentiated multilayers of the TR146 cell line. Infection assays were performed as described previously [47]. Prior to infection, *C. albicans* strains were grown in YPD at 30°C overnight and then diluted to OD_600_ = 0.2 in fresh YPD, followed by a new incubation at 30°C for approximately 4 h. Cells were harvested by centrifugation, washed three times with 1x PBS and finally resuspended in 1x PBS. 2×10^6^ *C. albicans* cells were added to each RHE. The infection assays were incubated for a maximum of 24 h at 37°C and 5% CO_2_. The release of lactate dehydrogenase (LDH) from epithelial cells into the cell-culture medium was measured to quantify the extent of epithelial cell damage. The CytoTox 96® non-radioactive cytotoxicity assay (Promega Corp., Madison, WI) was used to measure the amount of LDH in each sample. The reaction was assayed at 480 nm using a Genios plate reader (Tecan U.K. Ltd.). One unit of LDH activity is equivalent to 1 µM formazan formed per reaction. Result shown were generated from three separate infections. Prior to sectioning and staining for light microscopy, RHE tissues were fixed in 4% (v/v) paraformaldehyde in PBS (pH 7.4), dehydrated in ethanol and embedded in paraffin wax. Sections were stained with Periodic Acid Schiff (PAS) reagent for visualization of fungal elements. Tissues were examined using a Nikon Eclipse 600 microscope.

### Construction of *C. albicans* mutants

For transformation of *C. albicans* we have used the lithium- acetate method as previously described [48]. Ura^–^ mutant strains were recovered by integrating the plasmid pCIp10 (*URA3*) into the *RPS10* locus [49], [50]. A parental strain was created by integrating pCIP30 (*URA3*; *HIS1*; *ARG4*) [51] into the *RPS10* locus of BWP17. pCIp10 and pCIp30 were kindly provided by A. Brown, Aberdeen. The *pTET-UME6* construct was excised with the restriction enzymes *Apa*I and *Pml*I from a pNIM1 derivative kindly provided by A. Bito, University of Salzburg [28] and transformed into *eed1*Δ mutant strains. For experiments including *pTET-EED1* we have amplified the *SAT1-pTET* fragment from pNIM1 and the *EED1* ORF from genomic *C. albicans* SC5314 DNA with overhanging oligonucleotid primers (see Table S6). Both PCR fragments were linked in a fusion PCR and transformed into *C. albicans*. *pTET-UME6* or *pTET-EED1* were integrated into the *ADH1* locus as described previously [52]. Transformants were selected on YPD with 200 µg/ml nourseothricin [53] and were verified by PCR and Southern Blot analysis. All oligonucleotid primers used in this study are listed in supplemental table S6.

### RNA isolation

For RNA isolation cells were harvested by centrifugation and resuspended in 400 µl AE buffer (50 mM sodium acetate, 10 mM EDTA). Next, 40 µl 10% SDS and an equal volume of phenol/chloroforme/isoamylalcohol was added. Mixtures were incubated at 65°C for 5 min, followed by an incubation at −80°C until they were frozen. After a second incubation at 65°C (until samples were thawn) the mixtures were centrifugated for 2 min at 12000×g. The upper liquid phase was transferred into a new reaction tube. After addition of 10% volume sodium acetate pH 5.3 and 1 volume 2-propanol, RNA was precipated for 30 min at −20°C. Samples were centrifugated for 10 min at 12000×g, supernantant were discarded and RNA pellets washed twice with 70% ethanol (prepared with RNAse free water). Finally, RNA was solved in RNAase free water. Qualities and quantities of isolated RNA were analyzed with an Agilent 2100 Bioanalyzer (Agilent Technologies).

### Transcriptional profiling

For transcriptional profiling we have used *C. albicans* oligo microarrays (Eurogentec, Seraing, Belgium). Sample RNA from RHE and plastic assays was labelled with Cy5 (GE Healthcare). These sample RNAs were cohybridized with a common reference (RNA from SC5314 grown in YPD, mid-log phase, 37°C, Cy3- labelled). Slides were hybridized, washed and scanned with Genepix as described [54]. Data normalization (LOWESS) and analysis was performed with Genespring 7.2 software (Agilent Technologies) as described previously [12], [54]. Microarray studies were done in three biological independent triplicates. Student's t-test was used to compare expression data from the triplicates to identify significant differences. Only differences with p≤0.05 were used as statistically significant. All microarray data are MIAME compliant and raw data have been deposited at ArrayExpress (http://www.ebi.ac.uk/microarray-as/ae). The accession numbers are E-MEXP-3085 for the RHE infection microarrays and E-MEXP-3083 for the plastic growth experiments.

### Quantitative RT PCR

For gene expression analysis, 100 ng of total RNA were used to perform quantitative RT-PCR with the One Step RT-qPCR Master Mix Plus for SYBR Green I Kit (Eurogentec, Seraing, Belgium). RT-PCR was perfomed on a Applied Biosystems 7300 Fast Real- Time PCR System (Applied Biosystems, Darmstadt, Germany). Expression was calculated by the ΔΔCt method as described previously [55]. Student's t-test was used to compare expression data from the triplicates to identify significant differences. Only with p≤0.05 differences were regarded as statistically significant.

### Growth on plastic surfaces

Strains were twice grown in YPD at 30°C overnight for semisynchronisation. Cells were washed with 1x PBS and 1×10^5^ cells/ml were added to RPMI1640 medium (PAA). This mixture was given on petri dishes or 6- well plates and incubated for 24 h at 37°C at 5% CO_2_ in the air. For microscopy, a Leica DM IL inverted microscope (Leica Microsystems, Wetzlar, Germany) was used. To analyse dynamics of growth on plastic via timelapse microscopy we have used the same environmental conditions and a Zeiss AxioObserver. Z1 fluorescence microscope (Zeiss, Göttingen).

### Microscopy

Differential staining of *C. albicans* during infection of host cells was done as described previously [8]. After fixation and washing with 1x PBS *C. albicans* cells were stained with rabbit anti-*C. albicans* polyclonal antibody conjugated with Alexa Fluor 560 (Invitrogen). Subsequently, human host cells were permeabilised with 0.5% Triton X-100 and Calcofluor White was added to stain *C. albicans* cells. Therefore, fungal elements of *C. albicans* outside of host cells were stained with the antibody and Calcofluor White while parts within the host cells were stained with Calcofluor White only. For staining of cells grown on glass coverlslips without human cells, *C. albicans* cells were fixed with 4% histofix for 30 min (Carl Roth, Karlsruhe, Germany), washed with PBS and stained with Calcofluor White prior to microcopy. Microscopy was performed with a Leica DM 5500B fluorescence microscope (Leica Microsystems, Wetzlar, Germany). Image acquisition and analysis was done using the Leica Application Suite Software.

## Supporting Information

Movie S1 SC5314 RPMI plastic 37C 5% CO_2_
(AVI)

Movie S2 eed1 RPMI plastic 37C 5% CO_2_
(AVI)

Table S1 Genes down-regulated in eed1 during RHE infection
(XLS)

Table S2 Genes up-regulated in eed1 during RHE infection
(XLS)

Table S3 Transcriptome plastic growth WT vs eed
(XLS)

Table S4 Transcriptome plastic growth eed1+pTET-UME6 vs eed
(XLS)

Table S5 Binding motifs in intergenic region upstream of EED1
(DOC)

Table S6 Oligonucleotide primers used in this study
(DOC)
