# Supplementary material for: The Candida albicans-Specific Gene EED1 Encodes a Key Regulator of Hyphal Extension
Source: PLoS One. 2011 Apr 7;6(4):e18394. doi: 10.1371/journal.pone.0018394 (PMC3075580; doi:10.1371/journal.pone.0018394)
Supplement: Table S5 — Binding motifs in intergenic region upstream of EED1 (DOC) [file pone.0018394.s007.doc]

**Table S3: Motifs in the intergenic region upstream of orf19.7561 (*EED1*)**

**Binding Motif Sequence Position Reference**

**for upstream of *EED1***

Nrg1 CACCCT -476 to -482 [21]

Nrg1 CCCCCT -962 to -968 [21]

Nrg1 ACCCCT -1112 to -1118 [21]

Efg1 CATTTG -1191 to -1197 [32]

Efg1 CATTTG -1712 to -1718 [32]

Efg1 CATTTG -1865 to -1871 [32]
