# Supplementary material for: The Candida albicans-Specific Gene EED1 Encodes a Key Regulator of Hyphal Extension
Source: PLoS One. 2011 Apr 7;6(4):e18394. doi: 10.1371/journal.pone.0018394 (PMC3075580; doi:10.1371/journal.pone.0018394)
Supplement: Table S6 — Oligonucleotide primers used in this study (DOC) [file pone.0018394.s008.doc]

**Table S2. Oligonucleotide primers used in this study.**

**for deletion of the *EED1* gene by SAT1-flipper**

**oligonucleotide primer sequence (5’ to 3’ direction)**

5' SAT-FLP veri CTACATCAATGAAATCCAGACAGTCGAG

3' EED1-SF veri CGGTAGAGGCGTAAACTGGCAGATACC

ADHp fwd GTCATGTGCACGGACAAGC

ADHt rev CCAGAAAGCATTCATCGCG

pNIM1 fwd CAGAAACGACAAACATGGACAAC

pNIM1 rev TGCTCTACACCTAGCTTCTGG

pTET veri agtcgagtttaccactccctatc

**for quantitative RT PCR**

**oligonucleotide primer sequence (5’ to 3’ direction)**

EED1-R1 TAGTGGTAATACCCAACGTG

EED1-R2 CTGATATTTGAAATTTTGGAAGCTTTTC

UME6-R1 TCTACTTCTAATCCAATGGTG

UME6-R2 TATCATTACTTGATTTTTTCCGAG

ECE1-R1 ATCGAAAATGCCAAGAGAG

ECE1-R2 AGCATTTTCAATACCGACAG

HWP1-R1 ATCAGCTCCTGCCACTGAAC

HWP1-R2 TGAGTGGAACTGATTCTAATGTAGTTG

ACT1-R1 TCAGACCAGCTGATTTAGGTTTG

ACT1-R2 GTGAACAATGGATGGACCAG

EFB1-R1 AGTCATTGAACGAATTCTTGGCTG

EFB1-R2 TCTTCATCAACTTCATCATCAGAACC

**for construction of *pTET-EED1* fusion**

**oligonucleotide primer sequence (5’ to 3’ direction)**

pNIM1-F1 gagatggagccgtcaaatatcc

pNIM1-F2 gacaatcttgattgggcatttg

CaNIMR1 AATAGTGGAAAAGCTTCCAAAATTTCAAATATCAGAAATTTGTTAAATTGA

Cacatggcatggatgaactata

CaNIMR2 CGGCCTGTTCCGTTTCTAATGTTGGATGTGTTAAATTGTCTTCTTTCCAT

Ctttgtcgactatttatatttg

EED1R5 CCAGATTTCCAGATTTCCAGAATTTAGATCTTTATTTGTATAGTTCATCC

ATGCCATGTGtcaatttaacaaatttctgatatttgaaattttggaagc

EED1F5 CAAATATAAATAGTCGACAAAGatggaaagaagacaatttaacacatcc
